# Physical activity guidance in the rheumatology clinic—what matters for patients with rheumatoid arthritis? A qualitative study

**DOI:** 10.1007/s00296-023-05466-4

**Published:** 2023-10-03

**Authors:** Tanja Thomsen, Mette Aadahl, Merete Lund Hetland, Bente Appel Esbensen

**Affiliations:** 1 Copenhagen Center for Arthritis Research, Center for Rheumatology and Spine Diseases, Centre for Head and Orthopaedics, Rigshospitalet, Copenhagen, Denmark; 2 Center for Clinical Research and Prevention, Bispebjerg and Frederiksberg Hospital, Frederiksberg, Denmark; 3 Department of Public Health, Faculty of Health and Medical Sciences, University of Copenhagen, Copenhagen, Denmark; 4 The DANBIO Registry, Center for Rheumatology and Spine Diseases, Centre for Head and Orthopaedics, Rigshospitalet, Copenhagen, Denmark; 5 Department of Clinical Medicine, Faculty of Health and Medical Sciences, University of Copenhagen, Copenhagen, Denmark

**Keywords:** Rheumatoid arthritis, Physical activity, Health professionals, Qualitative study

## Abstract

Higher proportions of patients with rheumatoid arthritis (RA) are physically inactive compared to the general population. A barrier to engaging in physical activity (PA) may be lack of consistent PA guidance from health professionals (HPRs). This qualitative study aimed to explore daily PA levels and the patients’ perspectives on current and future PA guidance from HPRs. We recruited 20 participants from five rheumatology departments in Denmark. The participants differed in socio-demography and clinical characteristics based on results from an earlier cross-sectional study. The interviews were conducted by telephone, online platforms or face-to-face. Data analysis was based on reflexive thematic analysis. Thirteen participants were female and mean age was 55 years. We generated four themes; (1) *Acceptance of the arthritis is a process*, which attributed to acknowledging RA as part of life before fully engagement in PA and exercise. (2) *Daily physical activity—motivation, barriers and benefits,* reflecting the participants’ preferred types of activities and motivations and barriers to PA. (3)* Physical activity guidance—your own responsibility?* This theme reflected how participants missed more comprehensive discussions with HPRs about PA. (4)* It is essential how, when and where physical activity guidance is provided*, referring to participants’ preferences for future PA guidance in the rheumatology clinic. The study emphasizes that an integrated focus on PA should be part of the rheumatology clinic. However, HPRs may need adequate training in how to guide and motivate patients with RA towards increased PA.

**Supplementary Information:**

The online version contains supplementary material available at 10.1007/s00296-023-05466-4.

## Introduction

Rheumatoid arthritis (RA) is characterized by symptoms that can interfere with regular engagement in exercise and physical activity (PA). PA is defined as any bodily movement produced by skeletal muscles that requires energy expenditure, whereas exercise is a subgroup of PA that includes planned, structured and repetitive activities, which aims to improve or maintain one or more components of physical fitness [1]. In addition to well-known health benefits on aerobic capacity and cardiovascular health [2, 3], PA has also been shown to reduce pain and fatigue and improve physical function and quality of life in patients with RA [2, 4–7]. However, the literature also shows that a majority of patients with RA are physically inactive, i.e., fewer meeting PA recommendations compared to the general population [8]. Barriers involve fear of joint damage, pain, fatigue and lack of knowledge that PA may in fact improve symptoms [9]. However, a recent review argued that a significant barrier may be that patients lack specific and adequate guidance from health professionals (HPRs) [10]. This is supported by a cross-sectional study [11] demonstrating that only 49% of 1061 Swedish patients with RA recalled having discussions on PA and exercise in the rheumatology clinic. Still, HPRs in rheumatology believe that PA is important for patients and an important aspect to discuss, although they also experience uncertainty about the specific guidance [12]. If receiving guidance from HPRs has an influence on motivation and engagement in PA in patients with RA, it seems obvious that this should be an integral part of rheumatology clinic. While the literature offers perspectives on PA guidance from the HPRs’ point of view, the patient perspective on this remains largely unexplored. Thus, to improve a possible integration of PA guidance as part of rheumatology clinic, the present study aimed to explore daily PA levels and perspectives on the current and future PA guidance from HPRs in patients with RA.

## Materials and methods

### Study design and philosophical stand

This study is part of the Joint Resources research programme [13–15], which holds an overall focus on PA and sleep in patients with RA. The PA part of the programme comprises a randomized controlled trial (RCT) [13], a register-based cross-sectional study [16], a cost-effectiveness evaluation [17] and this qualitative study following up on patient experiences on PA.

The epistemological position of the study is dual. The study is grounded in constructivism [18] as we acknowledge that interactions between researcher and patients are social constructions based on individual experiences. In addition, we accept that neither patients nor researchers can be separated from their previous knowledge on lifestyle guidance in the rheumatology clinic. In addition, the authors adopt an epistemological position of pragmatism [19] as this qualitative research addresses practical solutions to inform future PA guidance to patients with RA. For transparency, the authors comprise four university academics with PhDs, all women. One author is a medical doctor with > 25 years of experience in rheumatology clinical work and research. Furthermore, three authors (nurse, physiotherapist and occupational therapist) share an overall research interest in PA and self-management.

### Participants and recruitment

We recruited participants based on results from an earlier register-based cross-sectional study, which aimed to identify socio-demographic and clinical characteristics of participants in a RCT focusing on reducing sedentary behaviour in patients with RA [16]. We used purposive sampling to ensure that participants differed in age, disease history (with and without co-morbidities), educational level, smoking status and PA levels (exercising 1–2 times/week versus primarily physically inactive). These criteria were sent to one rheumatology outpatient clinic in each of the five regions in Denmark and nurses invited four eligible patients from each clinic to participate. If interested, patients were contacted by first author (TT) and details about study purpose and interview were discussed.

### Data collection

The individual semi-structured interviews were conducted by TT from September 2020 to March 2021. Due to the COVID-19 pandemic, they were conducted either by telephone, by online solution or face-to-face meeting at the rheumatology outpatient clinic. The first, second and last author collaborated on developing an interview guide (online resource) that sought to capture the participants’ experiences of daily PA levels and PA guidance in rheumatology clinic. TT collected information on participants’ self-reported socio-demography, clinical status and lifestyle before recording. The duration of the recorded interviews was on average 47 min (range 34–64 min).

### Data analysis

The interview recordings were transcribed verbatim using pseudonyms to preserve participants’ anonymity and the transcripts were entered into NVivo (Version 12, QSR International Ltd, Melbourne, Australia). The data analysis followed the six-phase reflexive thematic analysis (RTA) by Braun and Clarke [20]. Our analysis focused on both semantic and latent levels, considering both what the participants described (i.e., content) and how they described it (i.e., language and meaning). Based on the six analysis steps [20], TT first read the interview transcripts multiple times to increase data familiarity. Initial codes were generated focusing on identifying dimensions on daily PA levels and PA guidance from HPRs. During the second round of coding TT finalized a more robust set of codes. By grouping codes that shared meanings into clusters reflecting overarching domains of PA experiences and perceptions, initial themes were developed. To ensure boundaries of themes and overall fit with study objectives, themes were refined, defined and validated through discussions between first and last author. These discussions served to ensure a more nuanced reading of data rather than to seek consensus of meaning. Finally, TT collated the results and identified appropriate examples of quotations.

Throughout the analysis process several of the criteria for demonstrating the quality of thematic analysis were addressed as suggested by Braun and Clarke [20]. This included checking interview transcripts against recordings for accuracy, giving equal attention to each interview in the process of coding and ensuring that as many participants as possible were represented in the quotations [20].

## Results

### Sample characteristics

Table 1 provides participant characteristics on a collective level (*N* = 20). Most participants were female, mean age was 55 years (range 22–77) and with an equal distribution of smokers versus non-smokers and physically active versus physically inactive patients. Fourteen participants preferred telephone interviews, two preferred online solutions and four preferred face-to-face meetings. For participant characteristic on an individual level, please, see Table 2.

**Table 1:** Participant characteristics (*N* = 20)

Gender	
Female	13 (65)
Male	7 (35)
Age, mean (SD)	55 (11.6)
Civil status	
Married/cohabiting	13 (65)
Unmarried/living alone/widow	7 (35)
Highest attained education	
Elementary school	2 (10)
Skilled worker education	8 (40)
Intermediate (3–4 years)	6 (30)
Long (> 4 years)	4 (20)
Occupation/working status	
Full time	5 (25)
Part time	4 (20)
Unemployed	2 (10)
Flex job	3 (15)
Age-related retirement	6 (30)
Physical activity levels	
Meeting the national physical activity recommendations^a^	9 (45)
Not meeting recommendations	11 (55)
Smoking status	
Smoker	8 (40)
Previous smoker	4 (20)
Never smoker	8 (40)
Medical treatment	
Biologic medication	12 (60)
Co-morbidity	
Participants with co-morbidity	11 (55)
Number of co-morbidities, median (range)^b^	2 (1–3)

Data are presented as numbers (%) unless otherwise stated

^a^Danish national recommendations for physical activity say that an adult should engage in physical activity of moderate to high intensity at least 30 min per day and should engage in physical activity of high intensity at least twice a week (for at least 20 min)

^b^Co-morbidities included cardio-vascular disease, chronic obstructive pulmonary disease (COPD), asthma, stroke, diabetes mellitus, osteoporosis, cancer and osteoarthritis

National recommendations were updated in June 2023 (after this study) and now say that an individual should move for at least 30 min/day so one gets out of breath easily, should do muscle strengthening activities at least twice a week and should limit the amount of time spent sitting

### Themes

We generated four themes related to participants’ PA experiences and perception of PA guidance from HPRs. Extracts of transcripts are provided below in the text as examples.

**Table 2:** Individual participant characteristics

Participant	Age (years)	Gender	RA duration (years)	Comorbidity	Meeting physical activity recommendations^a^
#1	43	Female	18	None	No; primarily LPA
#2	75	Female	4	COPD, heart disease and asthma	No; primarily LPA
#3	65	Female	14	Stroke	No; primarily LPA
#4	47	Male	7	Diabetes	No; primarily sedentary
#5	36	Female	3	None	Yes; exercising regularly
#6	53	Male	10	None	No; primarily sedentary
#7	57	Female	4	Diabetes	No; primarily LPA
#8	45	Female	13	None	Yes; exercising regularly
#9	71	Female	16	Osteoporosis	No; primarily LPA
#10	41	Female	4	None	Yes; exercising regularly
#11	64	Male	12	Diabetes and asthma	No; primarily sedentary
#12	44	Female	16	None	Yes; exercising regularly
#13	68	Male	8	Skin cancer and COPD	No; primarily LPA
#14	55	Female	11	Osteoporosis	Yes; exercising regularly
#15	22	Female	4	None	Yes; exercising regularly
#16	65	Male	8	Osteoarthritis	Yes; exercising regularly
#17	37	Male	5	None	No; primarily LPA
#18	64	Male	15	Diabetes and osteoarthritis	Yes; exercising regularly
#19	71	Female	32	None	Yes; exercising regularly
#20	77	Female	43	Heart disease and asthma	No; primarily sedentary

*COPD* chronic obstructive pulmonary disease, *LPA* light physical activity

^a^Danish national recommendations for physical activity say that an adult should engage in physical activity of moderate to high intensity at least 30 min per day and should engage in physical activity of high intensity at least twice a week (for at least 20 min)

National recommendations were updated in June 2023 (after this study) and now says that an individual should move for at least 30 min/day so one gets out of breath easily, should do muscle strengthening activities at least twice a week and should limit the amount of time spent sitting

#### Theme 1. Acceptance of the arthritis is a process

When reflecting on daily PA patterns, ‘acceptance’ was recurrently mentioned, attributing to acknowledging RA as part of life. Participants described how they had neglected the arthritis the first years after onset and fought hard to maintain the same roles as usual, including fighting to keep the same intensity of daily PA in their lives, but not always successfully. They described that actual accepting the disease was an important mental step before they could fully engage in adapting and adjusting daily exercise and PA. The duration of this process could vary considerably:

“*I used the first nine years to deny that I have this disease. I did everything else than what they told me at the rheumatology clinic. I could not accept it. I was convinced that it was a mistake” *(INT 8)

The same participant later stated:

*“I realized that I could not run from this disease. I have now accepted it as part of my baggage. I know how to live with it, so my everyday life is as good as possible” *(INT 8)

This change of attitude to circumstances led to changes of the participants’ PA levels and performance. They highlighted adjustments of exercise and PA habits to the capability of their physique and mental well-being, e.g., replacing physically strenuous activities with activities easier on their joints. Examples of this included replacing running with walking or biking or splitting up the daily long walk into two.

“*I had to reinvent how to use my body. Earlier, when my feet hurt, that limited me in EVERYTHING! Now, my feet might hurt, but I can still move. I just go biking instead of running or walking” *(INT 18)

Beneath this acceptance of the arthritis and adjustments of activity patterns, there seemed to be an insist in participants not to let the arthritis decide how to live their lives. For instance, participants described how determined they were to run or bike, even on days with considerable levels of pain and fatigue. They had to control the arthritis, not the other way around.

“*I don’t want arthritis to stop me in what I like to do. Of course, it does stop me to a certain degree, but I am so will-powered, it will never stop me” *(INT 10)

In addition, another:

“*I have always known that it should not be my arthritis deciding what I can do. So, I have just been keeping myself active” *(INT 15)

#### Theme 2. Daily physical activity—motivation, barriers and benefits

Although with different intensities and motivations, participants described PA at some level in their daily routines. The types and intensities of activities varied from strengthening the joints, household chores, daily walks, bicycle rides, to high-intensity organized exercise, such as running, cycling in a club and tennis. Of leisure-time activities, walks (with the dog), dancing and water gymnastics were popular. It was obvious that PA did not necessarily mean exercise and sports. It was also about keeping busy throughout the day.

“*It is important for me to stay active. Regardless of pain, I move as much as I can, all the time, cleaning my house, fixing the roof and working in my garden. I do not just sit*” (INT 7)

Being physically active and busy was seemingly a natural part of life. Another participant stated:

“I *have always felt, even back from childhood, that it is important to be and stay physically active. Otherwise, your life will sooner worsen. The biggest chance for a reasonably good life is to continue moving*” (INT 3)

However, there were still participants struggling with motivation for exercise and PA of any intensity. Seemingly, because it had never been of great value to them. They often moved with low intensity (short walks, light garden work etc.). Besides lack of motivation, RA symptoms played a major role in many participants’ PA engagement. Participants described difficulties in performing and maintaining sport activities, e.g., rowing and skiing, because of pain, fatigue and fear of joint damage. In addition, comorbidities such as chronic obstructive pulmonary disease (COPD), diabetes and osteoporosis were mentioned as key barriers to PA.

Nonetheless, while there were differences in participants’ descriptions of PA levels, intensities, motivations and barriers, they consented on the RA-related and general health benefits of PA, for instance on fatigue and mental well-being. In addition, they experienced that symptoms were actually reduced if they overcame their fatigue and/or pain and went through with exercise or PA plans.

“When my joints hurt, it can be extremely difficult to get going, but when my whole body gets moving, I don’t feel pain the rest of the day. And my energy gets better, and the fatigue disappears a bit” (INT 8)

#### Theme 3. Physical activity guidance—your own responsibility?

The theme depicts participants’ experiences of current PA guidance in the rheumatology clinic.

A central topic within this theme was the lack of standardized and consistent use of PA guidance in rheumatology clinic. Participants’ experiences with this matter were often initiated by own curiosity and insistence, e.g., asking HPRs about PA and exercise, actively seeking information about PA on the Internet, in Facebook groups and from the Danish Rheumatism Association. Information needs were linked to exercise and (team) training opportunities and tips for appropriate exercises. In some cases, participants had received this information through written material (pamphlets) from the rheumatology clinic. In general, participants missed more comprehensive and detailed discussions with HPRs about their lifestyle.

“*I wish that the staff would ask me of other things as well…I feel a bit left on my own. They should ask me about my life and my day, not making it all about my swollen joints, pain location and how I respond to the medicine.” *(INT 7)

In addition, another:

“*It is over so quickly when I visit the rheumatologist. You need to be finish before getting started. There is no room for any other questions” *(INT 19)

However, participants emphasised that if discussions about PA were actually initiated in consultations, they received support to increase PA levels. This included recommendations to move as much as possible within the limits of acceptable joint pain. Especially younger participants (< 60 years of age) described how they had initiated and brought up questions about PA and potential related constraints during consultations with their rheumatologist or nurses. The same age group was also the one, who described that they often had found relevant solutions to physical activity limitations through different websites or together with HPRs. Yet, participants had also experienced an opposite and more insecure guidance from HPRs, including insecurity about specific PA recommendations and being advised to spend the day sitting/lying down in case of pain. When expanding this, nurses were seen as the HPR participants would go to with queries not related to medical treatment. The participants expressed security and familiarity with the rheumatology nurses.

“*Every time you get a new doctor, it starts all over. I prefer to go to the nurses, who I know better. I talk to them about many things and I am not afraid to ask. I am always treated well” *(INT 16)

#### Theme 4. It is essential how, when and where physical activity guidance is provided

This theme depicts the participants’ preferences and wishes for future PA guidance in rheumatology clinic. This includes factors, such as timing, content and location. The participants agreed that lifestyle guidance should involve more than pamphlets.

”*What I have missed the most, is information. But the question is whether I was ready for this in the beginning where a bomb had just exploded on me. Surely, there were pamphlets, but I just threw them away. A real offer of lifestyle guidance should be more than a stack of pamphlets”. *(INT 8)

The above quote indicates that the timing for PA guidance is essential as participants would prefer the guidance after an initial period of time adjusting to the arthritis. There were different views on group-based versus individual guidance. Participants emphasized the social benefits they would gain by group-based exercise guidance, but they also expressed disadvantages to groups. These included seeing the arthritis as a personal matter or not wanting to identify oneself with other patients with arthritis. This participant stated:

“*I have not accepted 100% that I suffer from this disease. I have not reached that point, where it would be okay for me to be in a group, where others would know about my condition. For me, that is very personal.” *(INT 15)

In addition, another:

“*I am frightened by the thought of being in a group with other people, who are sick. That is certainly not me. I cannot identify myself with that*.” (INT 12)

There was a more mutual acknowledgement among participants on tailoring the PA guidance to the needs of the individual.

“*It would be so good if someone asked me how MY situation is, what I can do myself to feel better, what would work for me, how often is possible for me, all such things. And it might need to be an ongoing service as things can change*.” (INT 14)

The above quote also presents the PA guidance being a continuing service in rheumatology clinic, conducted by request from the individual person rather than a standardized service, as the need for support in lifestyle changes can vary.

“*If you need to be there, let’s say four times a year, and you don’t have anything to discuss or have a need for it, it would be a waste of healthcare resources in my opinion. I would prefer an opportunity to ask for a consultation if needed*” (INT 12)

Those participants who preferred PA guidance in the clinic as per request from the individual patient were predominantly those participants who had lived with RA for a long period of time (> 10 years). For types of needs and topics to address during PA guidance, participants proposed knowledge-sharing, reviewing motivation, opportunities, barriers and specific actions to take to increase PA levels. As previous, the participants likewise recommended the rheumatology nurse as the HPR with whom they would feel most comfortable when discussing lifestyle matters.

“*To establish some sort of an alliance with a nurse, who knows you, and by all means, the same one over time, that would be the best*” (INT 8)

In addition, physiotherapists were mentioned as potential delivers, especially by those participants preferring actual exercise classes. Regarding location, most participants would prefer PA guidance in conjunction with already planned consultations in the rheumatology clinic. At least, to start with.

“*I would prefer that it started out in the outpatient clinic. At one point, when you have a good starting point, then you could start up closer to where you live*.” (INT 9)

## Discussion

The purpose of this qualitative study was to explore daily PA levels and perspectives on clinic-based PA guidance in patients with RA. Our analysis revealed that patients may need to accept their arthritis as part of life before adjusting PA levels to new physical capabilities. It was apparent that integrating PA and daily movement into everyday life was more natural for those participants considering PA as an inherent value. Central barriers to engaging in PA were related to the arthritis and to co-morbidities. Finally, the analysis suggested that PA guidance in the rheumatology clinic was often a low-prioritized and rather inconsistent subject in the consultations. The participants called for PA guidance to be an integrated part of the rheumatology clinic, preferably a certain time after diagnosis, as an individually tailored continuing service with the same nurse throughout.

Our results depict several aspects in the lives of patients with RA that influence the patients’ daily PA behaviours. In the following we address some of the identified barriers. It is well-established knowledge that PA levels decrease after being diagnosed with arthritis, and patients report lack of motivation, fatigue, pain and functional limitations as barriers to PA and exercise [9, 21, 22]. This is supported by our study. However, a notable finding in our study was how acceptance of the arthritis was described as a significant mental step in performing and matching activities to new capabilities. The patients, who had struggled to come to terms with the arthritis diagnosis, described how they had fought to retain the same life roles and activities up to 9 years after diagnosis, and often had failed in this attempt. This is in line with an earlier qualitative interview study exploring disease acceptance in patients with RA [23]. Here, a five-stage acceptance process was introduced, including a ‘resistance phase’, where patients would be reluctant to accept the imposed limitations on daily activities, keep on doing the same activities, and in many cases give up on it. The process [23] also includes an ‘integration phase’, in which patients would begin using practical strategies to change daily routines to respect new limitations. This is in accordance with our findings, where patients explained how they had accepted the need to adjust their exercise and PA habits to the capability of their muscles, joints and mental well-being. As such, if acceptance of the arthritis plays a major role in PA and exercise behaviour in patients with RA, identifying whether the patient has reached some sort of acceptance of life circumstances may need to be considered by HPRs in promoting PA in patients.

Another potential barrier we need to address is the lack of consistent PA guidance from HPRs. No participants in our study described PA guidance as part of a standard offer in the rheumatology clinic. Rather, the topics of PA and exercise were addressed sporadically and mostly as per request from the individual patient. Noteworthy, it was the younger part of our participants, who requested information and discussions about lifestyle, including PA, with the rheumatology HPRs. This implies that in future PA guidance HPRs should be particularly attentive to the older group of patients and take the initiative to inquire about PA. The lack of systematic PA guidance was also reported by patients with inflammatory arthritis from the United States in a cross-sectional study, where only few respondents reported that exercise recommendations were addressed by HPRs [21]. In our study, the participants experienced the quality of the guidance as varied; from feeling supported and met with competent advice to a more insecure and deficient guidance from the HPRs. Insecurity especially arose in relation to the specific PA recommendations for patients with arthritis or to the safety of joints during high-intensity exercise. From the HPR’s point of view, similar uncertainties were indicated by rheumatologists, nurses and physiotherapists in a Dutch cross-sectional study about general attitudes towards PA and guiding patients with RA [24]. Here, more than half of the HPRs expressed a need for further education on the promotion of PA [24], which is in line with other evidence documenting lack of knowledge and confidence in PA promotion, and thus, educational needs among rheumatology HPRs [25, 26]. For instance, while 52% of physiotherapists from a recent cross-sectional study reported advising patients with RA to engage in PA, up to 62% never recommended the appropriate PA guidelines [26]. As such, and supported by a recent review about PA promotion in RA [10], an essential barrier to engaging in PA and exercise in patients with RA, may be the lack of consistent and specific directions from HPRs. However, as indicated in the recent review [10] and in the present study, sufficient PA guidance may not only be a matter of providing patients with knowledge about general PA recommendations and the health benefits of engaging in PA. Most patients with RA understand the health benefits but may need validation of their PA behaviour and specific guidance from HPRs regarding frequency, intensity and types of activities matching their individual needs.

In continuation, participants in our study gave us insight into their specific wishes and preferences for PA guidance in the future. Overall, the participants acknowledged that addressing PA and exercise should be integrated as part of treatment in rheumatology clinic. Furthermore, they suggested to tailor the PA promotion to the life circumstances of the individual patient, as a continuing service with the same nurse over time. Though, we need to acknowledge that the preference for a continuing, individually tailored service was especially expressed by those of our participants who had lived with and managed RA for over 10 years. There may be different needs among patients who have been recently diagnosed. The specific suggestions from study participants are integrated in a current implementation of individual PA guidance in four rheumatology outpatient clinics in the Capital Region of Denmark. In addition to results from our own research [13, 14], this implementation effort is based on accumulated evidence of PA and exercise on health benefits in patients with inflammatory arthritis [3], and is inspired by a proposed implementation model for facilitating long-term sustainability of PA in patients with RA [27]. The model includes training of HPRs to deliver brief PA advising during routine patient visits based on specific guidelines and current PA recommendations [27]. As such, supporting HPRs to embed PA guidance in rheumatology clinic may be an important approach in reducing physical inactivity and promoting health in patients with inflammatory arthritis [28]. This is not a new notion in the rheumatology community. In light of the vast evidence on PA and inflammatory arthritis and suggested frameworks for implementation, a recent editorial from *Rheumatology Advances in Practice (2023)* stated that ‘it is time to push the change’ to optimize the delivery and use of PA as an efficient management strategy in rheumatic diseases [29].

Addressing some methodological considerations of our findings is needed. First, there are several strengths. The study examines the patient perspective on current practitioner-led PA guidance through qualitative methods, which ensured rich and deepened descriptions of patient experiences. In addition, the study included a national sample of patients with RA from five Danish rheumatology outpatient clinics, which strengthens the transferability of our findings. Another strength is the research team’s combined background covering both a MD, a physiotherapist, a nurse and an occupational therapist with extensive clinical and research experience in medical treatment, disability, PA and self-management in rheumatology. This allowed for nuanced interpretations of data.

Second, there are important limitations that must be acknowledged as well. Although we initially instructed nurses in the rheumatology outpatient clinics about the inclusion criteria (age, education, smoking status, PA levels, co-morbidities), we cannot be certain how these criteria were interpreted in the actual clinics. Thus, the sample may not be that varied as aimed for. For instance, the sample includes a higher proportion of participants with a relatively low educational level. Another limitation related to selection and inclusion of participants is that the nurses, who informed and invited patients to the study may have invited those patients with whom they had the best communication and contact. Linked to this notion, we recognized many descriptions during data analysis that were related to the value of a good contact with the rheumatology nurse. A final limitation is that 14 of 20 interviews were conducted as a telephone interview including an inability to observe body language and facial expressions. In addition, we did not test the interview guide before the first interview.

In conclusion, this qualitative study expands our understanding of motivation and barriers of engaging in PA and exercise in patients with RA. The study also gives insight into current experiences and wishes for future PA promotion in the rheumatology clinic from a patient perspective. Patients specified that PA guidelines are not used consistently in rheumatology clinic and HPRs seem to lack knowledge of the central concepts and recommendations for PA in patients with RA. This calls for further efforts for improving and implementing PA as part of rheumatology clinic, including building up capacities in HPRs. It is time to push the change indeed!

### Supplementary Information

Below is the link to the electronic supplementary material.

Supplementary file1 (PDF 129 kb)

## Data Availability

Most data relevant to the study are included in the article or uploaded as supplementary information (interview guide). For further questions regarding data or reuse of data, please contact the corresponding author (tanja.thomsen@regionh.dk)
