# Supplementary material for: Physical activity guidance in the rheumatology clinic—what matters for patients with rheumatoid arthritis? A qualitative study
Source: Rheumatol Int. 2023 Oct 3;44(1):181–9. doi: 10.1007/s00296-023-05466-4 (PMC10766747; doi:10.1007/s00296-023-05466-4)
Supplement: Supplementary file 1 — Supplementary file1 (PDF 129 kb) [file 296_2023_5466_MOESM1_ESM.pdf]

Opening
Tell me about your everyday life including family, work, housing, hobbies etc. What does a typical day look like?

1) Everyday life and lifestyle
How do you consider your lifestyle in general (physical activity, diet, smoking and alcohol), and in relation to your arthritis?

2) Daily physical activity
Tell me about your daily movement, including physical activity and exercise. How much do you move during a typical day? – and how much time do you spent sitting?

3) Physical activity guidance in the healthcare system
Tell me about the information you have received in the rheumatology outpatient clinic about RA and lifestyle, including physical activity and exercise

4) Future physical activity guidance – content and structure
How would you imagine an offer of physical activity guidance from HPRs in the rheumatology clinic?

Supplementary questions

1. **Everyday life and lifestyle**
- What are your experiences with physical activity/daily movement/exercise?
- What are your experiences with other lifestyle factors such as diet, smoking and alcohol consumption?
- And what do you think of your lifestyle in relation to the arthritis?

2. **Daily physical activity**
- How much do you value physical activity and exercise? For instance, is it a natural part of life for you and your family/partner?
- Would you like to be more physically active than now? If so, what kind of activity would you like to do? What makes it difficult?
- How do you consider physical activity and exercise in relation to the arthritis?

3. **Physical activity guidance in the healthcare system**
- How was the guidance carried out?
  - By whom?
  - Where?
- If no memory or experience of guidance – what have you missed of information and guidance about physical activity and rheumatoid arthritis?

4. **Future physical activity guidance – content and structure**
- For instance, if you knew that it was possible to get a personal 1:1 session with a health professional three times a year, where you would discuss your daily physical activity? What would you talk about?
- For instance, what are your thoughts about participating in a supervised exercise program? Individual or in groups? And if you needed to attend a class 1-1½ hours 2-3 times a week.
- What would hinder you to attend either individual sessions with a HPR or the exercise classes? And on the other hand - what would act as facilitating factors?
- Of health professionals, who would you prefer to meet during these sessions? – a nurse, a physiotherapist, an occupational therapist, others?
- In what settings would you prefer the sessions? At the rheumatology outpatient clinic, as a health service in your municipality, or other places?

Interview guide to patients with RA (N=20)
Physical activity guidance in rheumatology clinic. Clinical Rheumatology. Thomsen T et al. Corresponding author: Tanja Thomsen, Center for Rheumatology and Spine Diseases, Rigshospitalet, Copenhagen. Mail: tanja.thomsen@regionh.dk
